# Crystal Chemistry of Carnotite in Abandoned Mine Wastes

**DOI:** 10.3390/min10100883

**Published:** 2020-10-04

**Authors:** Sumant Avasarala, Adrian J. Brearley, Michael Spilde, Eric Peterson, Ying-Bing Jiang, Angelica Benavidez, José M. Cerrato

**Affiliations:** 1 Department of Earth and Planetary Sciences, University of Tennessee, Knoxville, TN 37916, USA; 2 Department of Earth and Planetary Sciences, MSC03 2040, University of New Mexico, Albuquerque, NM 87131, USA;; 3 Center for Micro-Engineered Materials, University of New Mexico, Albuquerque, NM 87131, USA;; 4 Department of Chemical and Biological Engineering, University of New Mexico, MSC 01 1120, Albuquerque, NM 87131, USA; 5 Department of Civil, Construction & Engineering, MSC01 1070, Center for Water and the Environment, University of New Mexico, Albuquerque, NM 87131, USA;

**Keywords:** uranium, crystal chemistry, carnotite, reactivity, carbon inclusions, abandoned mines, electron energy loss spectroscopy (EELS)

## Abstract

The crystal chemistry of carnotite (prototype formula: K_2_(UO_2_)_2_(VO_4_)_2_·3H_2_O) occurring in mine wastes collected from Northeastern Arizona was investigated by integrating spectroscopy, electron microscopy, and x-ray diffraction analyses. Raman spectroscopy confirms that the uranyl vanadate phase present in the mine waste is carnotite, rather than the rarer polymorph vandermeerscheite. X-ray diffraction patterns of the carnotite occurring in these mine wastes are in agreement with those reported in the literature for a synthetic analog. Carbon detected in this carnotite was identified as organic carbon inclusions using transmission electron microscopy (TEM) and electron energy loss spectroscopy (EELS) analyses. After excluding C and correcting for K-drift from the electron microprobe analyses, the composition of the carnotite was determined as 8.64% K_2_O, 0.26% CaO, 61.43% UO_3_, 20.26% V_2_O_5_, 0.38% Fe_2_O_3_, and 8.23% H_2_O. The empirical formula, (K_1.66_ Ca_0.043_ Al(OH)^2+^_0.145_ Fe(OH)^2+^_0.044_)((U_0.97_)O_2_)_2_((V_1.005_)O_4_)_2_·4H_2_O of the studied carnotite, with an atomic ratio 1.9:2:2 for K:U:V, is similar to the that of carnotite (K_2_(UO_2_)_2_(VO_4_)_2_·3H_2_O) reported in the literature. Lattice spacing data determined using selected area electron diffraction (SAED)-TEM suggests: (1) complete amorphization of the carnotite within 120 s of exposure to the electron beam and (2) good agreement of the measured *d*-spacings for carnotite in the literature. Small Differences between the measured and literature *d*-spacing values are likely due to the varying degree of hydration between natural and synthetic materials. Such information about the crystal chemistry of carnotite in mine wastes is important for an improved understanding of the occurrence and reactivity of U, V, and other elements in the environment.

## 1. Introduction

Uranium (U) mining operations during the 1900s resulted in thousands of abandoned uranium mines in the United States. More than 4500 abandoned U mine waste sites have been identified in the Western United States, of which more than 500 are located on the Navajo Nation [1–4]. The Blue Gap/Tachee Claim 28 mine waste site in Northeastern Arizona was abandoned after mining operations ceased during the 1960s and was reclaimed in the early 1990s. Despite the reclamation, elevated concentrations of U and vanadium (V) have been measured in the water sources proximate to the Claim 28 mine waste site. Dissolution of uranyl vanadate (U-V) minerals found at this site was identified as the source of these elevated concentrations [3,5,6]. Although the reactivity and occurrence of these uranyl vanadates have been studied in detail, understanding of their crystal chemistry is still limited.

Uranyl vanadate minerals are abundant and important constituents of many uranium deposits. The co-occurrence of U and V as primary ore minerals such as carnotite (K_2_(UO_2_)_2_(VO_4_)_2_·3H_2_O) and tyuyamunite (Ca(UO_2_)_2_V_2_O_8_·(5–8)H_2_O) has been reported in mines from numerous locations in the world including the Southwestern U.S., South Dakota, Southwest China, Southern Jordan, Korea, and Australia [7–15]. For example, carnotite and tyuyamunite are abundant in limestone deposits of New Mexico occurring in the limestone-hosted U-ore bodies of the Grants mineral belt. These uranyl vanadates were formed as a product of schoepite reaction (UO_2_)_8_O_2_(OH)_12_·12(H_2_O) with Ca and K, under alkaline conditions created by limestone-buffered groundwaters [16–18]. Solubility of such uranyl vanadates has been identified as an important mechanism controlling the reactive transport of U and V from mine wastes in Northeastern Arizona [5,19]. Although the U and V-bearing mineral in these solubility studies was assumed to be carnotite, the determined solubility constant (K_eq_ 10^−44.81^) was 12 magnitudes lower than that of synthetic carnotite (K_eq_ 10^−56.38^) at circumneutral pH [5]. Therefore, to further assess the reasons for this discrepancy, additional research to characterize the chemical composition, crystallinity, and structure of the U-V bearing minerals in the mine waste is necessary.

The crystal chemistry of synthetic uranyl vanadates has been studied. The general formula, M^n+^_1/n_UO_2_AO_4_·xH_2_O represents the stoichiometry of various uranyl vanadate minerals: ‘M’ is a cation that can be either mono-, di-, or tri-valent elements of groups I and II in the periodic table; and ‘A’ can be P, As, S, or V [20–24]. For example, several uranyl vanadate minerals have structures that are comprised of [(UO_2_)_2_(V_2_O_8_)]^2–^ sheets and mono, di, or tri-valent cations [25–27]. The V_2_O_8_ groups in the [(UO_2_)_2_(V_2_O_8_)]^2–^ sheets are two tetragonal pyramids joined by a common edge. Recent studies also reported the co-occurrence of U and V as nano clusters of U-V oxide [28]. Although most uranyl vanadates consist of V_2_O_8_ groups that are connected to UO_7_ pentagonal bipyramids by a shared edge, these minerals Differ in hydration capacity, stoichiometry, bond distance, and bond angles [21,29–31].

Differences in hydration capacity and interlayer occupancy of cations in uranyl vanadates causes changes in structural arrangements and bond distances that in turn affect the thermodynamic solubility and crystal chemistry of these minerals [32,33]. Quantification of interlayer cations and water in such minerals can be challenging due to water loss and interlayer cation migration [33,34]. While the crystal chemistry of synthetic uranyl vanadate minerals, such as carnotite, has been widely explored, this information may not directly translate to the behavior of those minerals found in the environment, which pose serious concerns, such as those in mine waste associated with abandoned U mines.

To date, limited attempts have been made to apply advanced analytical techniques to investigate the crystal chemistry of carnotites occurring in the environment. Characterization of such natural minerals can be particularly challenging considering: (1) their occurrence as complex solid solutions instead of pure phases [35]; (2) varying degrees of crystallinity from amorphous to fully-ordered crystalline materials [5]; (3) the fine-grained nature of U-bearing minerals; and 4) their intergrown occurrence with other minerals, with variable degrees of hydration. The recent discovery of the mineral vandermeerscheite (K_2_[(UO_2_)_2_V_2_O_8_] 2H_2_O – P2_1_/*n*, *a* = 8.292(2), *b* = 8.251(3), *c* = 10.188(3) Å, β = 110.84(3), V = 651.4(4) Å^3^, and Z = 2) with Different crystal structure, but an ideal composition identical to carnotite (K_2_[(UO_2_)_2_V_2_O_8_] 2H_2_O (P2_1_/*c*, *a* = 19.2802(9), *b* = 7.0781(4), *c* = 5.3805(8) Å, β = 96.753(8), V = 729.2(1) Å^3^, and Z = 2) [27] further complicates the analysis of uranyl vanadates. In addition, uranyl vanadates typically also occur at concentrations that are below the detection limit of most bulk characterization techniques [36].

The objective of this study is to use various solid characterization techniques to investigate the crystal chemistry of the uranyl vanadate, carnotite that occurs in mine wastes from Northeastern Arizona. Results from this investigation provide new insights on the occurrence, structure, and chemistry of carnotite found in mine wastes that are fundamental to understanding complex mine waste behavior during weathering processes. Furthermore, this information will serve as a critical component in formulating an effective reclamation strategy for abandoned mines. Thus, promoting longevity and sustainable use of the land and its resources.

## 2. Materials and Methods

### 2.1. Materials

Mine waste samples were collected in November 2015 from Claim 28 mine, an abandoned U mine waste site in the Blue Gap/Tachee chapter, Navajo Nation, Northeastern AZ. The mine waste solid samples at this site were collected from the surface, within a 0.2 km radius of an erosional channel that cuts down through the soil overburden that was used to remediate the mine waste pile. The location of the mine waste samples was very similar to that used by Blake et al. (2015) [3]. Chemical characterization of these mine waste samples in Blake et al. (2015) suggested that U is present at concentrations <1 wt % [3]. Mine waste samples used in this investigation were present as rock fragments at the site, emitting very high gamma radiation (>10 mRad), high enough to be categorized as ore samples. These radiation measurements were made using a well-calibrated Ludlum Model 19 MicroR Geiger counter. Fragments from these mine waste rock samples were analyzed using a variety of spectroscopic, microscopic, and diffraction techniques.

Uranium ores in the Blue Gap/Tachee region occur in tabular and lenticular sandstone units of the Upper Cretaceous age Rough Rock Sandstone member of the Toreva formation. These U ores represent a much younger deposit of the arkosic sandstone unit formed by a less extensive transgression of the Cretaceous sea in the Northern Black Mesa that form the uppermost units of the Toreva formation [37]. The major mineralogical components of these deposits include quartz, potassium feldspar, clays, and uranyl vanadates [3,37,38].

### 2.2. Electron Microprobe Analysis

Small fragments (1.5 cm in size) of the mine waste rock samples were embedded in epoxy; the surface was ground flat and polished using successive grits of silicon carbide and alumina with a final polish using 0.05 μm alumina, suitable for electron microprobe analyses. Samples and standards were coated with a 150 nm thick layer of gold, rather than carbon, in order to measure carbon quantitatively. Additionally, scanning electron microscopy (SEM) imaging was conducted on epoxy-embedded mine waste fragments, to better understand the occurrence and distribution of the uranyl vanadates in the mine wastes.

X-ray mapping and quantitative analyses were acquired on a JEOL (Tokyo, Japan) 8200 electron microprobe equipped with 5 wavelength spectrometers (Electron Microbeam Analysis Facility in the Department of Earth and Planetary Sciences and Institute of Meteoritics, University of New Mexico, Albuquerque, NM, USA); automation of the microprobe and data reduction was carried out using Probe for EPMA(TM) (v.12.8, Probe Software, Inc., Eugene, OR, USA). Operating conditions for x-ray mapping were 15 kV accelerating voltage and 30 nA beam current. An initial set of quantitative analyses, including carbon, were carried out on gold-coated samples at 15 kV accelerating voltage, 10 nA beam current, and beam diameter of 5–10 μm. Natural mineral standards were used for calibration; kaersutite and hematite from the Harvard Mineralogical Museum [39] were used for O, Na, Al, K, Ca, and Fe; dolomite and olivine mineral standards from the Smithsonian Institution [40] were used for C, Mg, and Si; SRM 663 Cr-V steel for V; and a natural sample of uraninite for U. Counting times were 60 s on peak for C and O, 40 s for V, and 20 s for all other elements; total background counting times were the same as on-peak times. Interference corrections were applied to V for interference by Ti, to C for interference by U, and O for interference by V [41] and a blank correction was utilized for Au N5-N6 overlap with C [42]. Area peak factors [43] were utilized to correct x-ray intensities of C for wavelength peak shift and shape changes. The resulting detection limit for C was 0.016 wt %.

A second set of EPMA analyses were conducted on a Blue Gap/Tachee carnotite sample, but without carbon in the suite of analyzed elements. This suite of analyses was conducted on a newly polished, fresh surface of the sample to avoid any vacuum drying that may have occurred with the sample from previous periods in the electron microbeam instruments. This sample utilized a carbon coating rather than gold to provide a conductive coating for quantitative analysis.

Tests were conducted prior to analyses to determine optimum beam conditions to minimize volatilization of structural H_2_O and K drift within the mineral structure. The volatilization of water and drift of particular elements such as K and Na away from the electron beam within the mineral structure are known phenomena in feldspars, hydrated minerals, and glasses due to heating under the electron beam [44]. Reducing the current density either by reducing beam power (lower accelerating voltage and/or lower beam current) or by use of larger spot size can reduce the generated temperature at the analysis spot [45]. The parameters for accelerating voltage, beam current, and beam diameter were varied in each test. Accelerating voltage was tested at 3 values, 20, 15, and 12 kV; beam current varied through values of 10, 5, and 1 nA; and spot sizes of 10, 5, and 1 micrometer were checked. With each combination, the element count rate for K, U, and O was continuously monitored for 6 min of dwell on the sample. In addition, after each test, the surface was examined with a secondary electron image (SEI) to look for evidence of cratering due to loss of H_2_O. The higher accelerating voltages and beam currents tended to leave visible pits in the carnotite after the 6-min test while the lowest beam energy and current did not leave a mark. The spot size was kept as small as practically possible due to the thin, lath-like nature of the carnotite and to avoid carbon inclusions. Ultimately, 12 kV, 1 nA, and 0–2 μm beam diameter was chosen with increased counting periods for major elements of 60 peak and 30 s on background (since trace elements were measured previously, they were omitted from the analytical scheme).

The probe for EPMA software also utilizes a time dependent intensity correction scheme to determine the count rate of “self-volatile elements” and extrapolates back to the count rate at time zero to correct for K loss and concomitant increase in the count rate of other major elements. However, this correction was inconsistent, with some TDI corrections upward while others were the opposite. Thus, the TDI did not result in any significant improvement to the K analysis results [33].

The suite of analyses at 12 kV was compared to those acquired at 15 kV, 10 nA, and 5–10 μm spot diameter. In both cases, K and O values were lower than expected, oxygen being significantly lower. For the second set of analyses, measured oxygen was removed from the analytical scheme and instead calculated by stoichiometry from the cations; H was then calculated by the Difference from 100% in order to estimate H_2_O. This method may overstate H_2_O if carbon inclusions are present within the analytical volume. However, it provides more reliable results for the analysis of the other element such as K, U, and V since the x-ray absorption correction for both O and H are then included in ZAF correction algorithm [46].

### 2.3. X-ray Diffraction (XRD)

Mine waste samples collected from Blue Gap/Tachee Claim 28 mine site were analyzed using a Rigaku (Tokyo, Japan) SmartLab powder x-ray diffractometer (Center for Micro-Engineered Materials, University of New Mexico, Albuquerque, NM, USA). The Rigaku SmartLab XRD is fitted with a Cu Kα x-ray source with 1D silicon strip detector (D/teX™) and Ni filter Bragg Brentano geometry with 2/3 degree incident slit. Mineral phases were identified using Rietveld refinements performed using the Jade™ (MDI) software package (v.7, Livermore, CA, USA). Due to the beam divergence resulting from the 2/3 degree S6 incident slit, beam spillover during data collection occurred at angles below 20° 2θ, causing clay fractions to be most likely underestimated. The analysis was conducted on a mine waste rock sample that was prepared by grinding together some of its smaller fragments using an alumina mortar/pestle. The ground sample was processed through 3 Different sample preparation techniques to concentrate the uranyl vanadate for improved diffractograms. The first pattern was collected on a randomly oriented mine waste powder sample prepared using standard procedures. The second pattern was measured on the suspended fraction of the ground powder, obtained by mixing the powder with deionized water and allowing the solution to settle overnight [47]. However, due to a preferred orientation of this sample, the analysis was repeated using a more randomly oriented suspended fraction, obtained by reducing the settling time to <30 s.

### 2.4. Focused Ion Beam (FIB) Sample Preparation

An ultrathin section of the uranyl vanadate was prepared for transmission electron microscope analysis using the focused ion beam in situ lift out technique following the procedure described by Abreu and Brearley 2010 [48]. The focused ion beam (FIB) sample was prepared using a FEI Quanta 3D Dualbeam® FEGSEM/FIB instrument (Electron Microbeam Analysis Facility in the Department of Earth and Planetary Sciences and Institute of Meteoritics, University of New Mexico, Albuquerque, NM, USA) equipped with an EDAX Apollo 40 SDD EDS system. A Ga^+^ ion beam voltage of 30 kV and beam currents ranging from 3 nA to 10 pA were used to perform sample extraction and final ion milling. A protective layer of Pt, 2 μm in thickness, was deposited on the surface of the sample to minimize ion beam damage during the sample preparation. After the initial cutting stage, the FIB section was lifted out with an Omniprobe 200 micromanipulator and transferred onto a Cu TEM half grid for ion milling to a final thickness of about 100 nm.

### 2.5. Transmission Electron Microscopy (TEM) and Scanning Transmission Electron Microscopy (STEM)

The TEM analysis (Electron Microbeam Analysis Facility in the Department of Earth and Planetary Sciences and Institute of Meteoritics, University of New Mexico, Albuquerque, NM, USA) was conducted on the FIB section and handpicked grains of the uranyl vanadate from the mine waste to examine its microstructure and composition at the submicron scale. The handpicked uranyl vanadate (yellow) grains 50–100 μm in size were taken from fragments of the mine waste rock sample and disaggregated under acetone using an agate pestle and mortar. A droplet of the disaggregated sample suspension in acetone was deposited on a holey carbon TEM grid and allowed to dry prior to TEM analysis. Both types of uranyl vanadate sample were studied using bright-field TEM imaging (BFTEM), selected area electron diffraction (SAED), and energy dispersive spectroscopy (EDS) using a JEOL 2010F FASTEM Field Emission Gun FEG/STEM instrument (Electron Microbeam Analysis Facility in the Department of Earth and Planetary Sciences and Institute of Meteoritics, University of New Mexico, Albuquerque, NM, USA). This instrument is equipped with a GATAN GIF 2000 and an Oxford AZTec EDS system with an ultrathin window XMax^N^ 80 mm^2^ SDD EDS detector. In addition, the FIB section was also studied using high-angle annular dark-field scanning transmission electron microscopy (HAADF STEM), full spectral STEM x-ray mapping, energy-filtered TEM imaging (EFTEM), and electron energy loss spectroscopy (EELS). The JEOL 2010F was operated at 200 kV for all analyses except for EFTEM imaging, which was performed at 197 kV. The EELS analysis was operated at an accelerating voltage of 200 kV and energy resolution of 0.7 ev.

### 2.6. Raman Spectroscopy

Raman spectroscopy was performed on uranyl vanadate grains 50–100 μm in size mounted on double-sided sticky carbon tape, mounted on a glass slide. The Raman measurements were made using a WITec Alpha 300R Confocal Raman microscope (Center for Micro-Engineered Materials, University of New Mexico, Albuquerque, NM, USA), utilizing a 532 nm laser. In addition to the Blue Gap Tachee (BGT) mine waste sample, a carnotite sample from the University of New Mexico (UNM) Mineral Collection (UNM 1249) was also run at the same time. The spectra from the BGT uranyl vanadate and the UNM carnotite sample were compared with carnotite spectra from the Mineralogical Society of America RRUFF Raman spectra database.

## 3. Results and Discussion

### 3.1 Raman Spectroscopy

**Figure 1.**
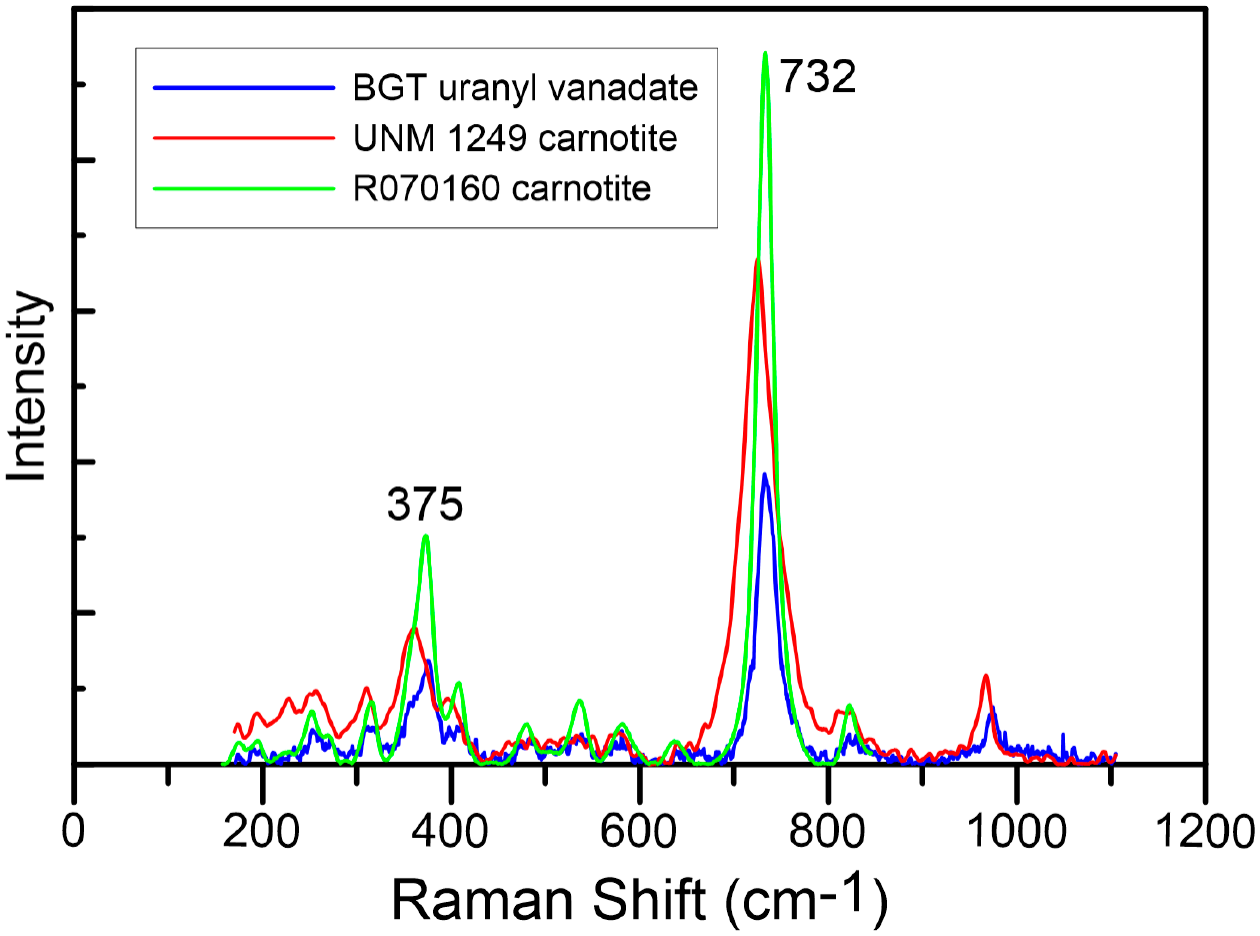
Raman spectrum (blue) for the BGT Claim 28 uranyl vanadate with Raman spectra for carnotite (R070160—blue) from the Mineralogical Society of America RRUFF Raman spectra database and a carnotite specimen (1249—red) from the University of New Mexico Mineral Collection. Each spectrum is scaled differently for comparison. The major peaks at 732 and 375 cm^−1^ are indicated. There is very good agreement between the Raman spectra from BGT and R070160, with the major peak at 732 cm^−1^, but the UNM carnotite shows a shift in the position of this peak to lower values. In comparison, the major peak for vandermeerscheite is at 745 cm^−1^.

The Raman spectrum of the uranyl vanadate specimen obtained from the BGT mine waste sample is shown in Figure 1, compared with spectra obtained from a carnotite (specimen 1249) from the UNM Mineral Collection and an example of a carnotite spectra (R070160) from the Mineralogical Society of America RRUFF Raman spectra database. The uranyl vanadate spectrum from BGT was lower intensity and noisier than the RRUFF R070160 spectrum but was in agreement in terms of the major peak positions. The most intense Raman shift peak was at 732 cm^−1^ for R0701060 and matched the position of the major peak in the BGT sample. This peak could be assigned to v_2_ and/or v_3_ stretching vibrations in the VO_5_ unit, as observed in previous vibrational spectroscopy studies of carnotite [49]. Likewise, the most intense peak for vandermeerscheite at 745 cm^−1^ could be attributed to the v_2_ or v_7_ stretching vibrations in the VO_5_ unit, very different from those observed in the case of carnotite [27], The UNM 1249 carnotite had a very similar spectral shape as both the BGT and R0701060, but shows a distinct spectral shift to lower values. We speculated that this shift might be the result of solid solution towards tyuyamunite, but we did not have compositional data for UNM 1249 to confirm this speculation. The second most intense peak at 372 cm^−1^ in the R0701060 spectrum was very close to the BGT peak value of 375 cm^−1^. This peak was representative of the V_2_O_2_ bending modes in the V_2_O_8_ units of uranyl vanadates that are characteristic to both carnotite [49] and vandermeerscheite [27], Conversely, the peak at 975 cm^−1^ that represents the v_1_ (VO_3_) symmetric stretching vibrations in the (V_2_O_8_) unit were more characteristic to uranyl vanadates such as carnotite and tyuyamunite, unlike in the case of vandermeerscheite where these peaks occurred at 970 cm^−1^ [27,49]. Similarly, the less intense peak at 825 cm^−1^ could be attributed to the v_1_ stretching of the (UO_2_)^2+^ unit, characteristic for carnotite [49] and not for vandermeerscheite where, the peak typically occurred at 820 cm^−1^ [27]. Therefore, the Raman data provided definitive evidence that the uranyl vanadate we studied at BGT was carnotite and not vandermeerscheite.

### 3.2. X-ray Diffraction (XRD)

**Figure 2.**
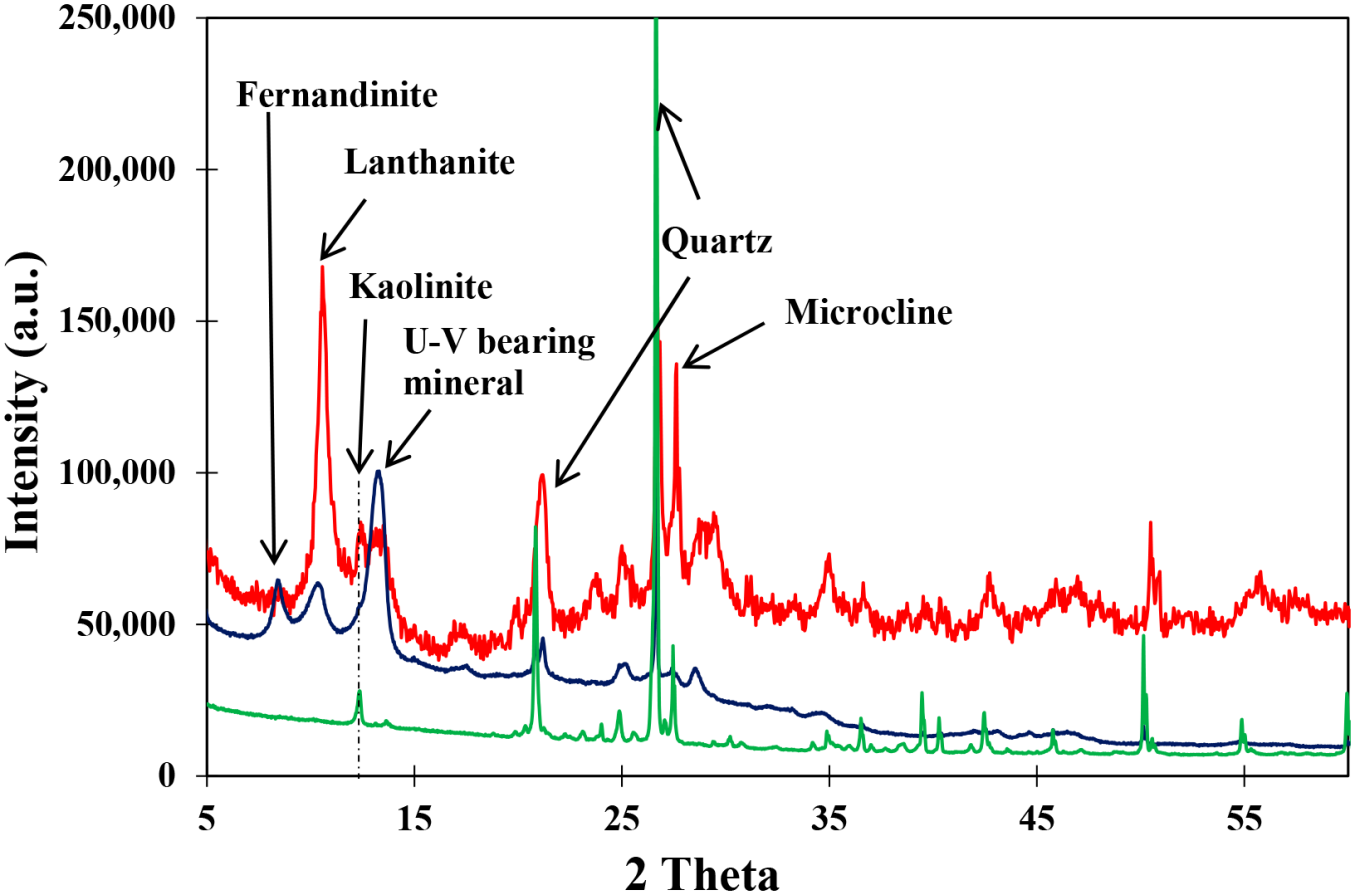
Powder x-ray diffraction pattern of Blue Gap Tachee mine waste. The green profile represents the diffractogram of the powdered mine waste as it is. The blue profile represents a diffractogram of the preferentially oriented mine waste sample obtained from the suspended fraction of a water separation technique with an overnight settling time. The red profile is a diffractogram of a randomly oriented mine waste sample obtained from the suspended fraction of a water separation technique with a settling time of <30 s.

Diffraction analysis of the Blue Gap/Tachee Claim 28 mine waste samples indicates that most diffraction peaks of the uranyl vanadate agreed with those of carnotite reported in the literature. However, in the ‘as-is’ sample, due to the relatively low concentration of U (<1 wt %) in the mine waste samples as reported previously [3], the occurrence of uranyl vanadate could not be detected in the diffractogram (Figure 2, green) due to its low abundance. The absence of identifiable diffraction peaks for uranyl vanadate could also be attributable to the fact that some of these uranyl vanadate minerals could be amorphous. In addition, the diffractogram was dominated by peaks from minerals such as quartz, kaolinite, and microcline, which were abundant in the host rocks [3]. However, x-ray diffraction analysis of the two samples where the uranyl vanadates were concentrated following the procedure described in the methods section did show the presence of carnotite. The diffractogram obtained on the sample prepared by overnight settling of the water-dispersed mine waste samples showed preferential crystal orientation. Only a few diffraction peaks were present in these diffractograms, but these had *d*-spacings that matched those of anhydrous carnotite [K_2_(UO_2_)V_2_O_8_], reported in the literature [21]. This result suggests that the uranyl vanadate in the mine waste sample had a similar structure to that of carnotite. In addition, the diffractogram also suggested the presence of fernandinite [CaV_8_O_20_·4H_2_O] and lanthanite [(REE)_2_(CO_3_)_3_(H_2_O)_8_] (Figure 2, blue). To overcome the preferential orientation issue and acquire a randomly oriented sample, another sample was prepared by reducing the settling time to <30 s. Diffraction results on this randomly-oriented sample (Figure 2, red) indicate that most uranyl vanadate peaks were in agreement with those of carnotite, better than those acquired for a slower settling rate. Although these results also suggest that the uranyl vanadate in the mine waste sample was carnotite, there were minor discrepancies between the natural and synthetic materials. Therefore, additional studies using electron microscopy techniques were necessary to better understand the crystal chemistry of the uranyl vanadate.

### 3.3. Electron Microprobe Analysis (Imaging, Qualitative, and Quantitative X-ray Mapping)

**Figure 3.**
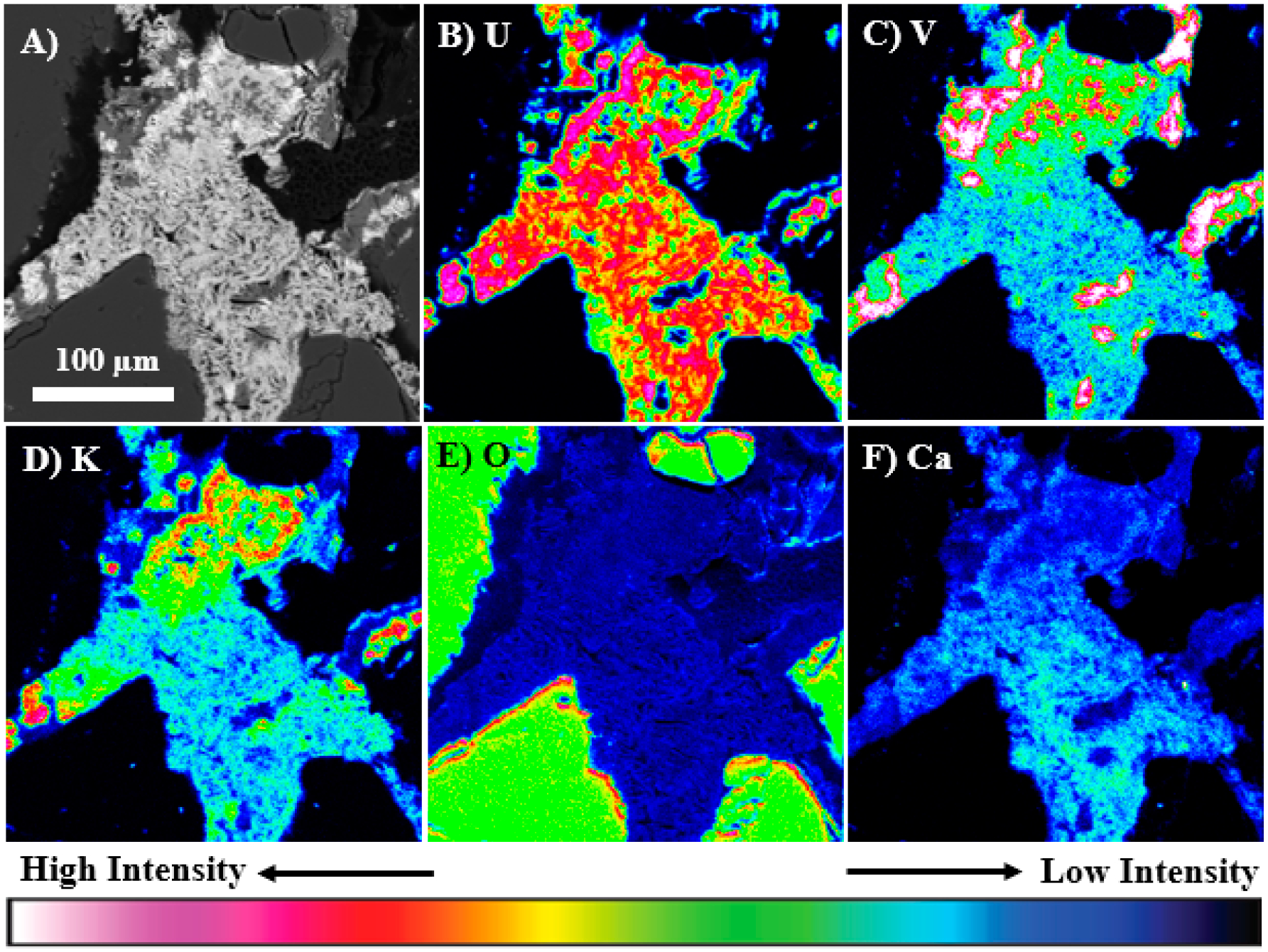
Qualitative wavelength-dispersive spectroscopy (WDS) electron microprobe x-ray maps of elemental distributions in an ore sample from mine waste from the Blue Gap Tachee site, showing complex correlation of U with V, K, Ca, and O. **(A)** Back-scattered electron image of the mapped area, (B) U M_α_ map, **(C)** V K_α_ map, (D) K K_α_ map **(E)** O K_α_ map, and **(F)** Ca K_α_ Map.

The electron microprobe X-ray mapping analysis of a U-bearing ore grain in the mine waste samples shows that the distribution of U was complex, but was commonly associated with V, K, Ca, and to a lesser extent O (Figure 3). The distribution of these elements was variable and patchy within this grain, but there were clearly regions such as that in the upper half of the ore grain that shows a strong spatial correlation between U, V, K, and O suggesting qualitatively that it had a composition that was consistent with carnotite, as indicated by the Raman and XRD analysis. However, the lower half shows lower K and V contents, but higher Ca, suggesting that an additional U-bearing phase such as to tyuyamunite may be present intergrown with carnotite. The apparent low concentrations of oxygen in the U-bearing phases based on the O x-ray map (Figure 3E) was due to the elevated concentrations of O in the surrounding quartz grains. In addition, a V-rich phase with low U content was associated with the U-rich phase; it is a fibrous V-Fe-bearing phase that has been previously discussed in Avasarala et al. (2017) [5], The two types of uranyl vanadate identified in the ore grain appeared to be intergrown and precipitated as secondary minerals on the surface of the V-Fe bearing phase.

**Figure 4.**
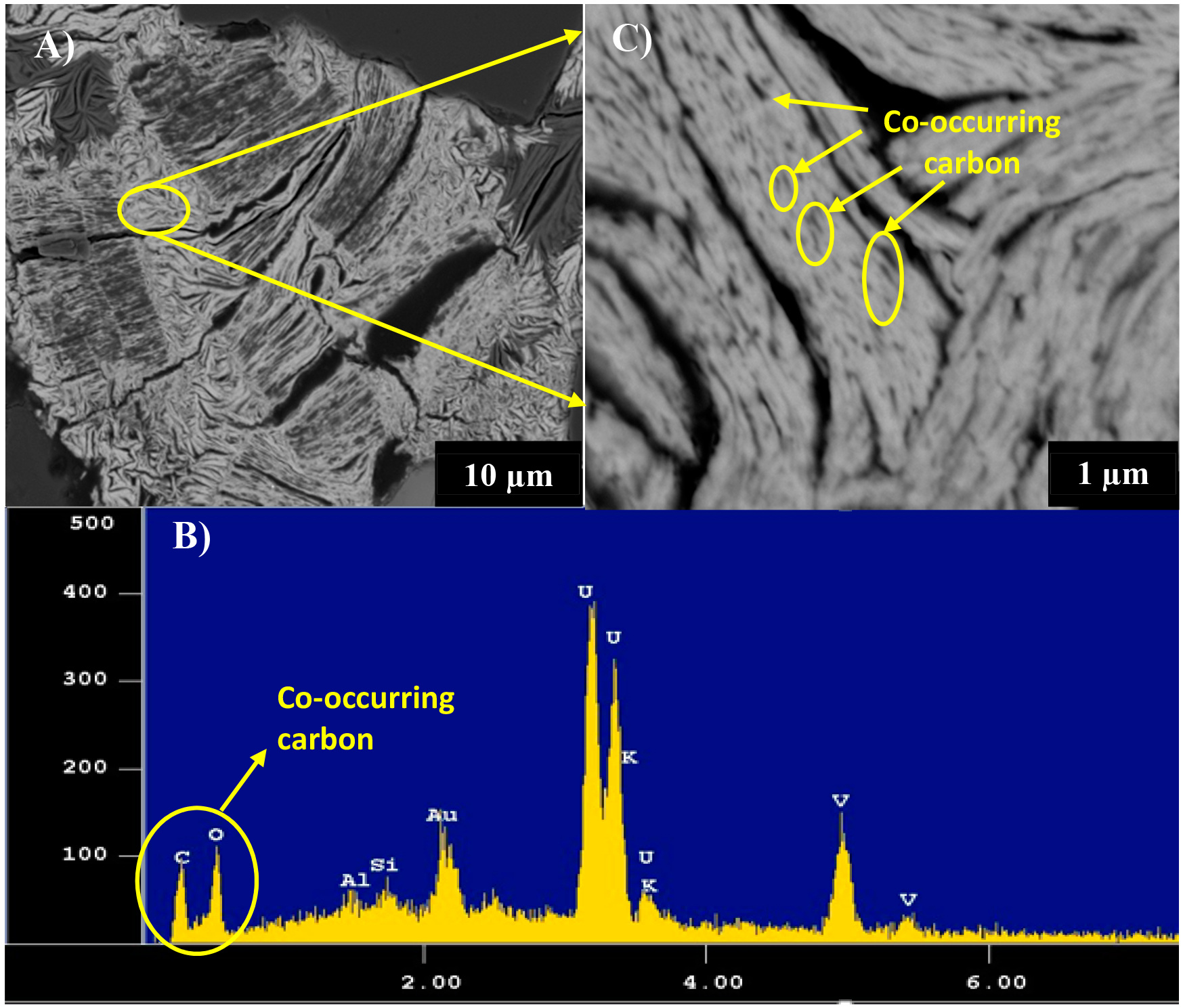
(A) **(A)** Back-scattered electron (BSE) image of complex intergrowth of U-V bearing mineral phases occurring in the Blue Gap/Tachee mine waste. **(B)** Energy dispersive spectrum (EDS) of the yellow circled area in image A showing presence of low Z inclusions. (C) Higher magnification BSE image showing the occurrence of submicron, dark low-Z inclusions (yellow circles) in the uranyl vanadate, which EDS indicates are carbon rich.

Back-scattered electron imaging of the uranyl vanadate shows that it contained numerous, submicron, low-Z inclusions distributed quite homogeneously throughout individual grains (Figure 4A,B). These grains show an alignment parallel to the elongation direction of the uranyl vanadate grains and ranged in size from 100 to 200 nm. Qualitative EDS analysis shows that these inclusions were rich in carbon (Figure 4B). Quantitative microprobe analysis of the identified uranyl vanadate was conducted on several different regions of the polished section of the epoxy embedded mine waste sample. Carbon was included in the analytical setup, based on the fact that the EDS spectra indicated that C was present. However, as discussed later, TEM studies show that this C was present in submicron inclusions that are disseminated throughout the uranyl vanadate grains. Therefore, after ignoring the included carbon from the quantitative microprobe analysis (4.8% C as elemental carbon) the elemental composition of the uranyl vanadate from the initial set of analysis was 4.45 wt % K_2_O, 0.53 wt % CaO, 61.43 wt % UO_3_, 0.04 wt % Na_2_O, 20.65 wt % V_2_O_5_, and 0.56% Fe_2_O_3_, at an atomic ratio of 1:2:2 for K:U:V (Table 1, (1)). These concentrations are very similar to that of carnotite [K_2_(UO_2_)_2_(V_2_O_8_)·1–3H_2_O] reported by Palache (1951), specifically for UO_3_ (62.26 wt % or 2 atoms per formula unit (apu)) and V_2_O_5_ (20.57 wt % or 2 apu); unlike in the case of K_2_O (10 wt % or 2 apu) that was almost double than those observed for carnotite occurring in mine waste [50,51]. The low K_2_O contents (K_2_O + Na_2_O + CaO = 5.02 wt % or 0.98 apu) could be attributed to migration of K from the electron beam excitation volume, despite using a broad beam of 10–15 μm, 15 kV and 10 nA for the analyses [33,52]. Stubbs et al. (2010) documented the analytical challenges of determining the compositions of Cu-bearing uranyl phosphates using an electron microprobe where copper, the interstitial cation, was highly mobile. It can be reasonably expected that K was probably even more mobile, based on its well documented mobility during EPMA analyses of feldspars and alkali-bearing glasses [44,53]. The issue of mobility was reasonably addressed when a second set of analyses were conducted at a lower beam power (12 kV and 1 nA), yielding 10.09 wt % or 1.9 apu K_2_O (Table 1, (3)). However, there is still a deficiency of cations in the site. Typically, EPMA analysis on hydrated minerals result in cratering due to heating within the electron interaction volume. A similar effect was observed during the higher beam power analysis; however, it was lost once the beam power was lowered for the second analysis. The 1.1 wt % deficit K however, could be attributed to the presence of hydronium ion (H_3_O^+^) substituting for K, similar to other large alkali-site minerals. This is in agreement with observations made in previous investigations that have associated alkali deficiencies in the jarosite-alunite group to hydronium displacement of K [54], Significant hydronium substitution results in totals of less than 1.00 apu for the cation totals at site-A in the jarosite-alunite group [55]. However, verification of hydronium requires NMR analysis, which is beyond the scope of this study.

In addition to the K-migration, consistent deficiency in totals was observed in the first set of EPMA analyses (90.65 wt %). These deficiencies can be attributed to water loss due to the formation of craters in the sample when exposed to higher beam power. However, on switching to a low power beam the wt % of water and K changed from 1.7 and 5.02 to 4.08 and 10.09, thus increasing the total analyzed cation content (Table 1). Taking these changes into consideration the new calculated empirical formula of the uranyl vanadate phase is (K_1.66_Ca_0_._043_Al(OH)^2+^_0.145_ Fe(OH)^2+^_0_._044_)((U_0.97_)O_2_)_2_((V_1.005_)O_4_)_2_·4H_2_O, which can be simplified to K_2_(UO_2_)_2_(VO_4_)_2_·2H_2_O at a 1.9:2:2 K:U:V ratio, very similar to the 2:2:2 ratio in the reference carnotite used in this investigation [50,51]. Therefore, the corrected EPMA analysis, coupled with the Raman and x-ray diffraction data provides strong evidence that the uranyl vanadate in the ore samples collected from the Claim 28 mine site is carnotite.

### 3.4. Scanning Transmission Electron Microscopy (STEM)

**Figure 5.**
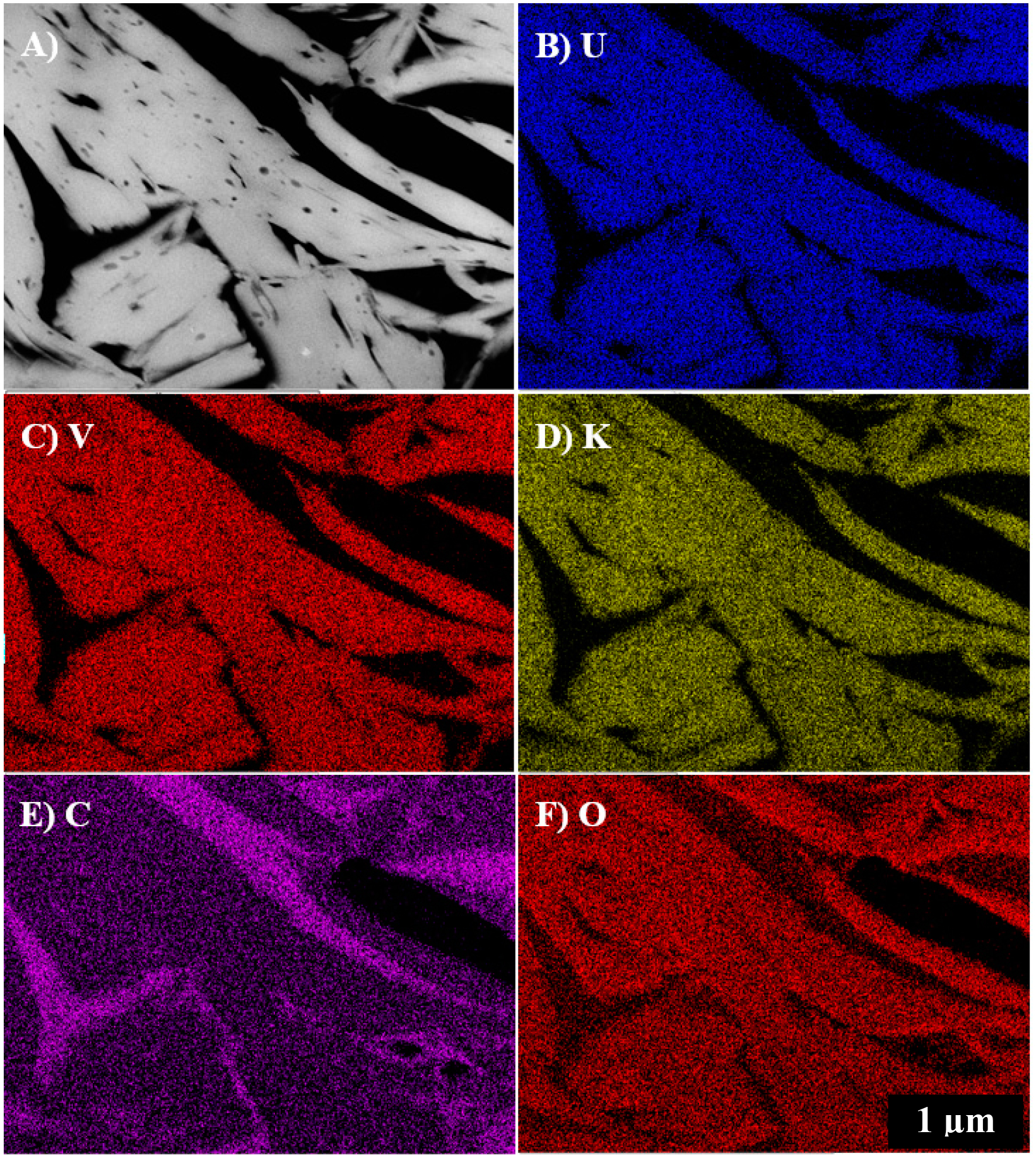
Scanning transmission electron microscopy (STEM) image and STEM EDS x-ray maps obtained from a focused ion bean (FIB) section of the uranyl vanadate from the Blue Gap Tachee mine waste. **(A)** High-angle annular dark-field (HAADF) image of the FIB section showing the elongate character of the uranyl vanadate crystals and the presence of significant intergranular porosity (dark—filled with epoxy) and the presence of low-Z inclusions (also dark) within the uranyl vanadate grains, **(B-F)** x-ray maps of **(B)** U M_α_, **(C)** V K_α_ **(D)** K K_α_, **(E)** C K_α_, and **(F)** O K_α_, showing the distribution of these elements within the uranyl vanadate grains. The carbon x-ray map shows the presence of C-rich inclusions within the uranyl vanadate grains and the presence of the embedding epoxy that has filled the intergranular porosity during sample preparation.

TEM and STEM analysis were used to investigate the crystal structure and microstructures of the uranyl vanadate, by studying both powdered fragments of the sample and a FIB section extracted from a grain that contained the low-Z inclusions. Bright-field TEM and electron diffraction analyses were performed on the samples prior to STEM imaging and STEM x-ray mapping. The BF-TEM imaging of the FIB section shows that the uranyl vanadate consists of crystalline, elongate, platy grains between 1 and 5 μm in size with a significant intergranular porosity. The grains often have their elongation direction parallel to subparallel. The STEM x-ray mapping shows that the uranyl vanadate grains are compositionally unzoned with a homogeneous distribution of U, V, K, and O throughout the individual grains (Figure 5). There was no evidence of intergrowths of any phase with a different composition, such as tyuyamunite, with the exception of the carbon-rich inclusions, which are discussed below.

**Figure 6.**
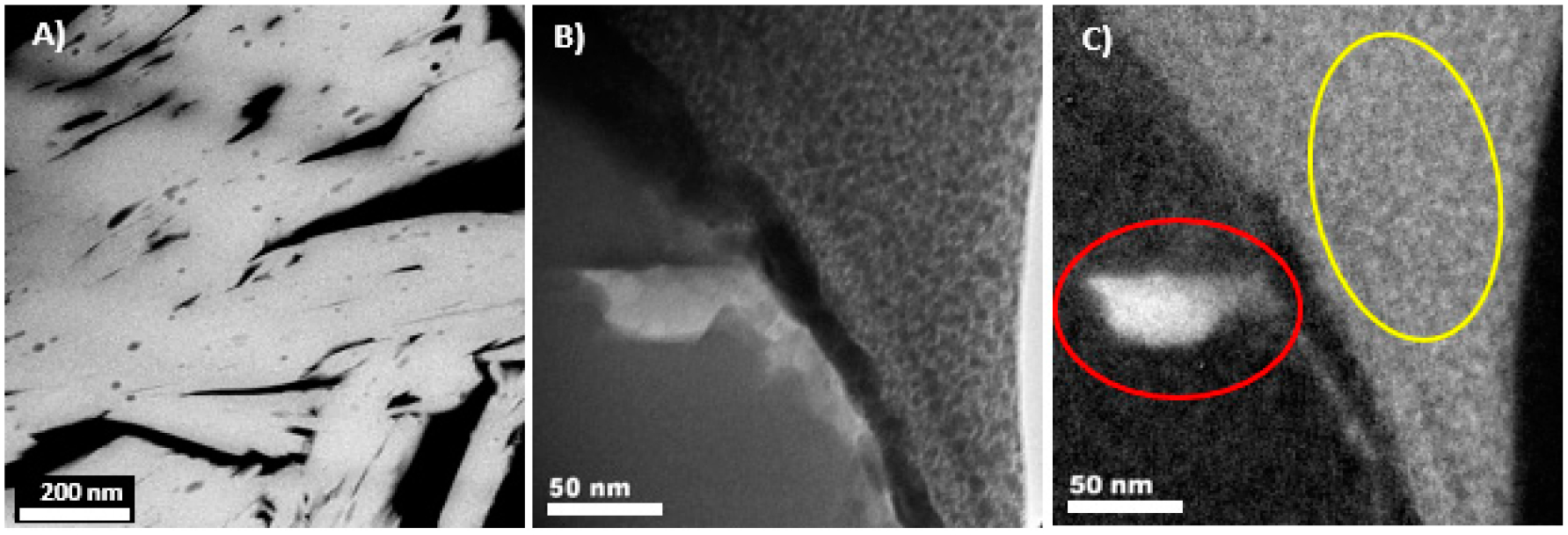
(A) **(A)** HAADF images in scanning transmission electron microscopy (STEM) mode showing the co-occurrence of C (dark inclusions) within the uranyl vanadate from the Blue Gap Tachee Claim 28 mine waste. **(B)** Bright-field TEM image and **(C)** energy filtered transmission electron microscopy (EFTEM) image carbon map confirming that the inclusion in the uranyl vanadate is carbon-rich. The area in red is a C-rich inclusion and the area in yellow indicates carbon retained in the organometallic Pt compound that is used to protect the FIB section from ion beam damage during sample preparation.

Dark-field STEM and EFTEM imaging were used to characterize the occurrence of carbon within the uranyl vanadate phase. In DF-STEM images (Figure 6A), distinct, submicron grains were distributed throughout individual uranyl vanadate grains. They ranged in morphology from sub-rounded to elongate grains that ran parallel to the elongation direction of the uranyl vanadate grains. Their sizes ranged in their longest dimension from 20 to 50 nm for the largest elongate grains. The EFTEM imaging using the C edge at 284 eV shows that the grains were indeed carbon-rich: no other elements were detected within these inclusions. A complicating issue with this analysis was that the abundant epoxy was present in the interstitial space between the uranyl vanadate grains. Therefore, our analysis was restricted to C-rich inclusions within individual uranyl vanadate grains and not due to the epoxy impregnation. The EFTEM image shown in Figure 6 is an example of a grain, which was just on the edge of a uranyl vanadate grain in a region that was thin enough to do EFTEM analysis.

**Figure 7.**
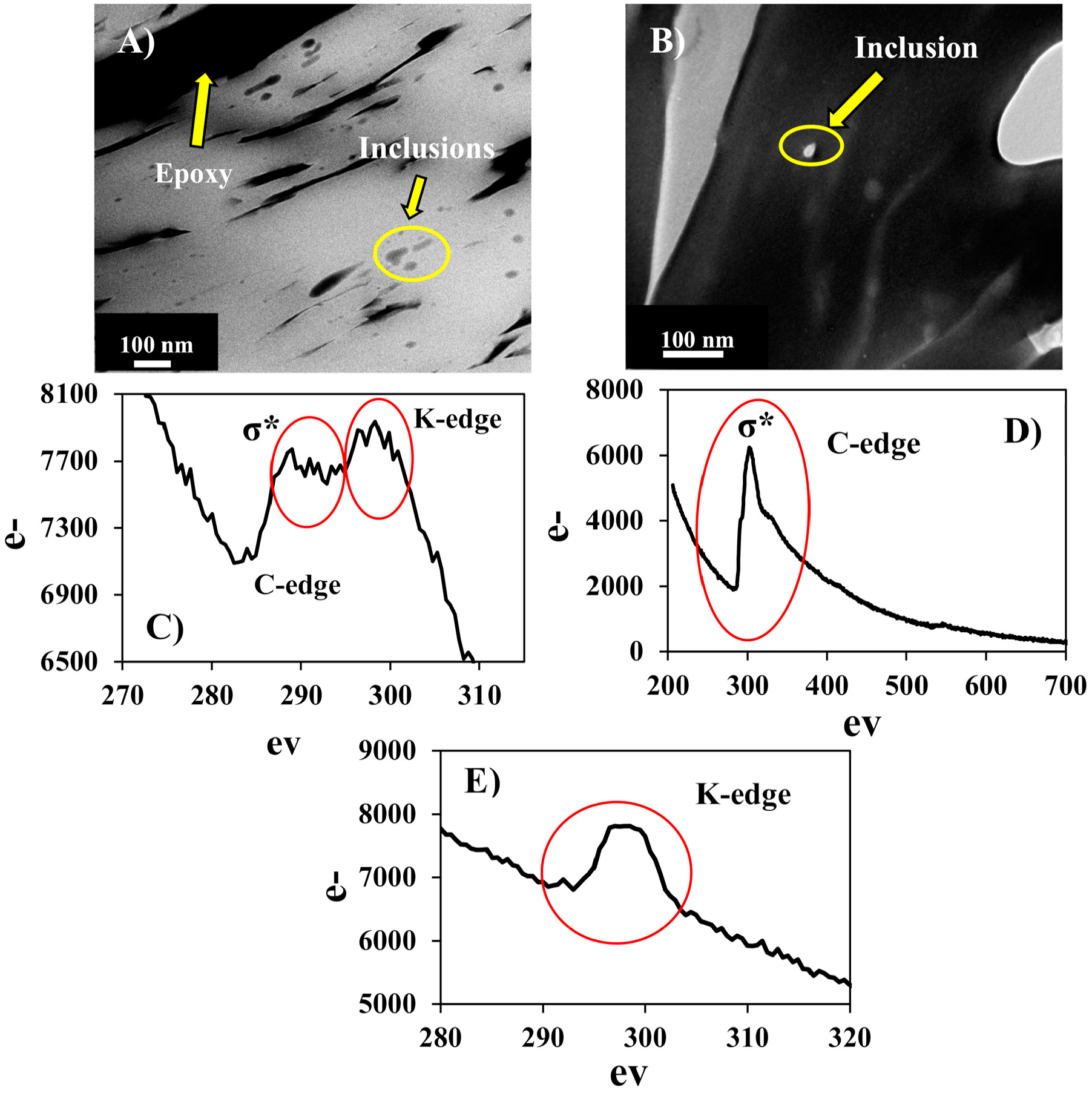
Electron energy loss spectroscopy (EELS) analysis of the carbon inclusions and epoxy. **(A)** HAADF scanning transmission electron microscopy (DF-STEM) image of the inclusions and epoxy (yellow arrows) in the focused ion beam (FIB) section of the uranyl vanadate. **(B)** Bright-field (BF)-TEM image of an inclusion that was analyzed using EELS. **(C)** EELS spectrum of the C inclusion showing the presence of C and K-edge (red circles). **(D)** EELS spectrum of the epoxy showing the presence of C-edge (red circle). **(E)** EELS spectra of the uranyl vanadate away from the inclusion showing the presence of only K-edge (red circle). The C L edge at 284 eV is missing demonstrating that the carbon is present only in the inclusions.

Electron energy loss spectroscopy (EELS) analyses were performed on several of the C-rich inclusions to determine their characteristics. Figure 7A shows a DF-STEM image of C-bearing inclusions in the region of the uranyl vanadate where the EELS analysis was conducted. An example of a specific inclusion that was analyzed is indicated in Figure 7B. The EELS spectrum from this inclusion shows the presence of a broad peak characteristic of σ* bonding with no evidence of a pre-edge π* bond signal at the C-edge. These features of the C edge were consistent with amorphous carbon. The analysis was complicated by the fact that the C K-edge at 284 eV was overlapped by the potassium L-edge, which occurred at an energy of 294 eV (Figure 7C). Although the electron beam was focused on the inclusion, to avoid beam damage, the electron beam was defocused resulting in an overlap with the uranyl vanadate and a contribution from the K hosted in it. In EELS spectra of amorphous and graphitic carbons, an increase in the π*/ σ* ratio, i.e., more π* bonding, occurred with an increasing degree of graphitic character [56], as the transition from amorphous to graphitized carbon (turbostratic or poorly graphitized carbon) and then crystalline graphite occurs. The strong sp^2^ bonding in graphite indicated in EELS spectra by a distinct, sharp σ* peak [57], whereas amorphous carbon, which does not contain sp^2^ bonding does not show the σ*. Therefore, (1) the broad and featureless shape of the σ* peak and (2) the small π*/ σ* ratio shows that the inclusion in the uranyl vanadate was amorphous C (Figure 7C). This amorphous C was likely organic matter from carbonaceous plant material that co-occurs with U in these deposits [38]. An EELS spectrum was also obtained at the C edge from the adjacent epoxy (Figure 7D), which was also amorphous. This spectrum was characterized by an intense σ* peak, unlike the inclusion spectra, which had a much broader σ* peak, demonstrating that the inclusions were not epoxy, but was indigenous to the uranyl vanadate. Several EELS spectra were also conducted on regions of the uranyl vanadate away from any obvious carbon inclusions (Figure 7E). These spectra showed no evidence of a C edge indicating that the carbon detected in the electron microprobe analyses must be dominantly present in carbonaceous inclusions and was not incorporated into the structure of the uranyl vanadate.

**Figure 8.**
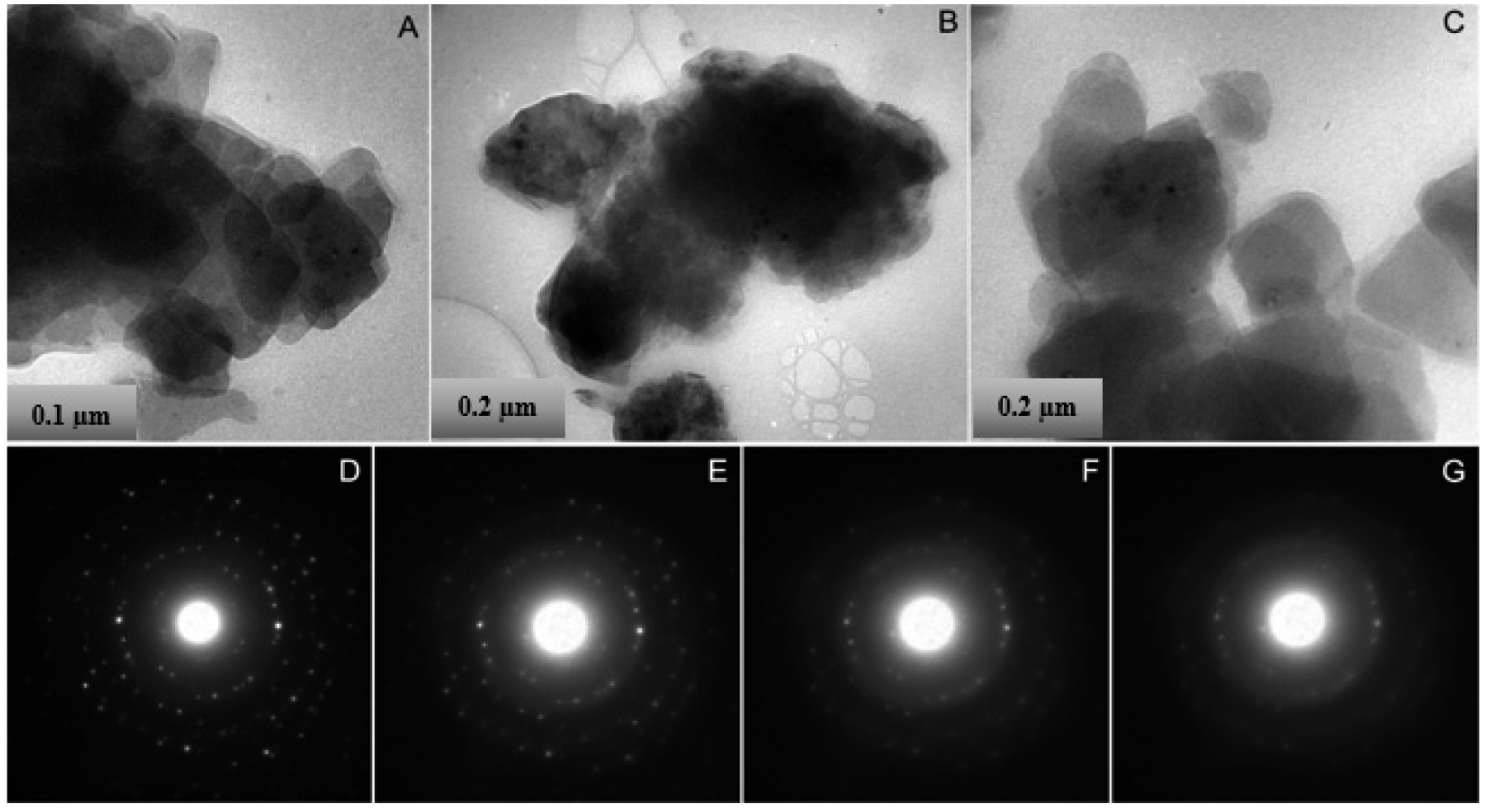
Bright-field TEM images and electron diffraction patterns of clusters of uranyl vanadate crystals from the Blue Gap/Tachee ore. **(A–C):** Bright-field TEM images showing the morphology and grain size of different uranyl vanadate clusters. The crystallites consist of plates with anhedral to subhedral outlines that are overlapping one another, parallel to the plane of the supporting holey carbon film. **(D–G):** sequence of ring electron diffraction patterns taken at time intervals of 20 s, showing the rapid amorphization of the uranyl vanadate due to electron beam irradiation. Discrete diffraction maxima disappear and are replaced by diffuse intensity consistent with an amorphous phase.

To supplement the powder XRD data, which suggest that carnotite is the dominant uranyl vanadate mineral in the mine waste, TEM studies were carried out on grain mounts of the fine-grained fraction of this phase from the mine waste. Electron diffraction data were also obtained from the FIB section of carnotite extracted from an aggregate in the mine waste sample. Specifically, the electron diffraction data was obtained from single crystals to confirm the identification of carnotite. Bright-field TEM imaging (Figure 8A–C) demonstrates that the uranyl vanadate occurred in the TEM grain mount as clusters of submicron crystals (Figure 8A–C). Several different clusters were examined in order to determine if there was any variability in the characteristics of the clusters. Morphologically, the crystals appeared to be platy, lying on top of one another and had subhedral shapes with weakly developed facets. Grain sizes ranged from <0.2 to 1 μm. Electron diffraction studies of individual clusters demonstrated that the uranyl vanadate was indeed crystalline, but rapidly amorphized during electron beam exposure. In order to obtain images and electron diffraction patterns of the sample, the grains were located on the TEM sample grid at low electron fluxes, with a highly defocused electron beam, giving very low illumination. After location of a grain cluster, an electron diffraction pattern was obtained at the same low level of electron irradiation. The patterns were ring patterns, but due to the limited number of crystals within the diffraction aperture, the rings were incomplete, but were still well-defined, nevertheless. To avoid excessive exposure of the grains to the electron beam, EDS analysis of the study area was only carried out after collection of the electron diffraction data. A sequence of electron diffraction patterns was then obtained for several different clusters of crystals. The electron diffraction patterns were taken under the same conditions, with only the time interval to collect and save the individual electron diffraction patterns (20 s) between each pattern (Figure 8D–G). The patterns show the progressive amorphization of the sample, indicated by the disappearance of diffraction maxima with high reciprocal lattice spacings and the development of diffuse electron diffraction intensity as rings within the patterns. From the first diffraction pattern obtained from each sequence of patterns, lattice spacings were calculated for each of the diffraction rings (Table 2).

In addition, electron diffraction patterns were obtained from several platy carnotite crystals in the FIB section. Due to the rapid amorphization of the samples, it was not possible to carry out tilting experiments to orient the individual crystals along zone axis. Nevertheless, each pattern contained well-defined diffraction spots; *d*-spacings were measured for these diffraction maxima. Table 2 presents a compilation of the *d*-spacings measured from both the ring patterns and from individual grains in the FIB section compared with *d*-spacing data for anhydrous carnotite, calculated using SingleCrystal software.

It is apparent from this comparison that, in general, there was very good agreement between the measured data and the calculated data. In addition, a number of diffraction maxima were absent from the measured *d*-spacing compilation, compared with the calculated spacings. Minor discrepancies between the measured and calculated values are to be expected for several reasons. The only crystal structure determination available for carnotite is for synthetic anhydrous carnotite reported by Sundberg and Sillén (1950) [58]. Anhydrous, synthetic carnotite was monoclinic with space group P2_1_/*c*. However, the uranyl vanadate occurring in mine waste was hydrated and, based on various studies of the Ca analog of carnotite, tyuyamunite, was likely to have variable states of hydration. Previous studies have shown that the progressive hydration of uranyl vanadates results in the expansion of the interlayer spacing, changing the dimensions of the unit cell [33,59]. Stern et al. (1956) showed that complete dehydration of tyunamunite results in the formation of metatyunamunite, a phase that Stern et al. (1956) suggested was structurally distinct from tyuyamunite, based on x-ray diffraction data [59]. Donnay and Donnay (1955) determined the structure of tyuyamunite and found that it is orthorhombic with space group P*nan*, compared with a monoclinic structure for anhydrous carnotite [60], Therefore, expansion of the unit cell due to variable degrees of hydration may account for the discrepancies between the calculated and measured values.

The absence of a number of diffraction maxima in the *d*-spacing compilation carnotite could be attributed to the fact that there is a preferential orientation of the clusters of particles on the TEM grid, with the plates of carnotite lying with their (001) planes parallel to the supporting holey carbon film. This was the exact same problem that was encountered with the XRD patterns produced by long settling times to make the powder XRD mounts. These patterns therefore only sampled a small volume of reciprocal lattice space.

Although there were minor differences between the measured and calculated patterns, which might be attributable to differences in the degree of hydration, the data for the uranyl vanadate occurring in Blue Gap/Tachee mine waste was consistent with carnotite. Importantly, the data also show that the carnotite was crystalline, but rapidly amorphized under the electron beam.

## 4. Conclusions

In this study, we showed by Raman spectroscopy and electron diffraction analysis that the uranyl vanadate at the Blue gap Tachee/Claim 28 mine site was carnotite. Carnotite is one of the major ore minerals in numerous, now abandoned uranium mines that present a significant environmental significance as mine wastes interact with source waters. Therefore, it is critical to understand their role in the reactive transport of U and V from mine waste piles [5]. Determining the occurrence, structure, composition, and crystallinity of these uranyl vanadates is essential to evaluate their behavior and inform future e orts for site remediation, resource recovery, and potential exposure reduction. Carnotite in the mine waste could occur as crystalline, elongated, and platy crystals, between 1 and 5 μm in size with a homogeneously distributed intergranular porosity. This intergranular porosity between the uranyl vanadate grains was filled with epoxy in the study sample, but the carnotite contained nanometer-sized elongate inclusions of amorphous carbon distributed homogeneously throughout individual grains. These inclusions were interpreted as relict grains of carbonaceous matter that was indigenous to the ore deposit. Excluding the carbon, the empirical formula of the uranyl vanadate was estimated to be K_1.9_(UO_2_)_2_(VO_4_)_2_·4H_2_O, with an atomic ratio 1.9:2:2 for K:U:V, which was very similar to that of the ideal formula for carnotite (K_2_(UO_2_)_2_(VO_4_)_2_·3H_2_O) 2:2:2 with minor discrepancies in the interlayer K content and H_2_O concentrations. Despite the deficiency in K between the synthetic and natural carnotite, Raman spectrum and the *d*-spacing data obtained by electron diffraction were consistent with carnotite. These results indicate that synthetic and natural minerals could experience potential discrepancies in crystal structure due to Differences in hydration that could affect their rates of reaction under environmentally relevant conditions. The knowledge obtained from this study and the integrated methodology used to obtain this information can be transcribed to other mine waste sites where uranyl vanadates are dominant [7–15]. This information will be especially helpful in developing effective reclamation strategies that will promote the conversion of abandoned and contaminated U mines to usable land.

## Figures and Tables

**Table 1.** Electron microprobe data for carnotite from the Blue Gap Tachee mine waste [49,50]. (1) 15 kV, 10 nA, 5–10 μm beam diameter, and measured oxygen. Average of 15 spots from 4 samples. The C in this quantitative analysis is treated as elemental carbon. (2) 12 kV, 1 nA, 1 μm beam, oxygen calculated from stoichiometry, and H by difference from 100%. (3) Reference carnotite [49].

Element	Element Weight%			Oxide	Oxide Weight%			Element	Formula Based on Stoichiometrically Estimated O (w/o H_2_O)		
	(1)	(2)	(3)		(1)	(2)	(3)		(1)	(2)	(3)
K	3.54	7.17	7.95	K_2_O	4.45	8.64	10.00	K^+^	0.881	1.677	1.887
Ca	0.37	0.19	0.46	CaO	0.53	0.26	0.66	Ca^2+^	0.088	0.043	0.105
Na	0.03	0	0.12	Na_2_O	0.041	0	0.16	Na^+^	0.012	-	0.046
U	52.20	51.12	52.58	Al_2_O_3_	-	0.81	-	Al(OH)^2+^	-	0.145	-
V	11.50	11.35	11.46	Fe_2_O_3_	0.56	0.38	0.55	Fe(OH)^2+^	0.022	0.044	0.02
				Sum A-site	5.581	10.09	10.82	Sum A-site	1.003	1.909	2.038
Al	-	0.43	-	UO_3_	62.72	61.43	62.26	U	2.046	1.948	1.935
Fe	0.41	0.27	0.40	V_2_O_5_	20.65	20.26	20.57	V	2.118	2.018	2.011
H		0.92	0.55	H_2_O	1.7	4.08	4.90	H	0.981	1.705	2.038
O	17.80	28.56	26.53								
Total without C	85.90	100.01	100.05	Total	90.65	95.86	99.50				
C	4.81		-	-	4.81		-				
Total with C	90.71				95.46						

**Table 2.** Measured *d*-spacings (in Å) based on electron diffraction patterns for the uranyl vanadate in the Blue Gap/Tachee mine waste compared to the calculated *d*-spacings for synthetic anhydrous carnotite (K_2_UO_2_VO_4_) [57], Lattice spacing data <2.5 Å has been omitted [57].

Calculated	h	k	l	Measured [57]
6.4626	0	1	1	6.427
6.3886	1	0	0	6.318
5.75				
5.0857	1	1	0	5.17
4.9478	1	1	1	4.92
4.68				
4.5438	1	0	2	4.58
4.3320	0	1	2	
4.2243	1	1	1	4.27
4.2015	0	2	0	
3.9969	1	1	2	3.95
3.8799	0	2	1	3.79
3.5620	1	0	2	
3.5104	1	2	0	
3.4640	1	2	1	3.41
3.2795	1	1	2	
3.2313	0	2	2	3.21
3.1943	2	0	0	
3.1858	1	2	1	3.13
3.1282	0	1	3	
3.1022	1	1	3	
3.0848	1	2	2	
3.0607	2	0	2	3.05
3.0606	2	1	1	2.99
2.9859	2	1	0	
2.8759	2	1	2	2.90
2.7170	1	2	2	2.71
2.7004	2	1	1	
2.6993	0	3	1	
2.6290	0	2	3	
2.6136	1	2	3	2.603
2.5885	2	2	1	2.596
2.5865	1	1	3	
2.5767	1	0	4	
2.5653	1	3	0	
2.5470	1	3	1	2.53
